# Let's Not Miss the Forest for the Trees: A Reply to Montefinese and Vinson's (2015) Commentary on Vieth et al. (2014)

**DOI:** 10.3389/fpsyg.2015.01984

**Published:** 2016-01-06

**Authors:** Harrison E. Vieth, Katie L. McMahon, Greig I. de Zubicaray

**Affiliations:** ^1^School of Psychology, The University of QueenslandBrisbane, QLD, Australia; ^2^Centre for Advanced Imaging, The University of QueenslandBrisbane, QLD, Australia; ^3^Faculty of Health, Institute of Health and Biomedical Innovation, Queensland University of TechnologyBrisbane, QLD, Australia

**Keywords:** lexical access, competition, semantic interference, picture naming, shared features, distinctive features

In Vieth et al. (2014a), we conducted three experiments to examine semantic relatedness effects in the picture-word interference (PWI) paradigm. According to the lexical selection by competition account of spoken word production, feature overlap between the target picture and related distractor word induces semantic interference. However, this account has been challenged by studies demonstrating semantic facilitation in the PWI paradigm (e.g., Costa et al., 2005; Mahon et al., 2007; but see Hutson and Damian, 2014; Sailor and Brooks, 2014; Vieth et al., 2014b). In Vieth et al. (2014a), we investigated whether some reports of semantic facilitation in PWI might be due to the influence of distinctive features, i.e., features that quickly distinguish a concept from other similar concepts, as previous studies had not controlled for this variable (e.g., Costa et al., 2005; Mahon et al., 2007; Sailor and Brooks, 2014). In Experiment 3, we observed semantic interference for distractor words denoting a non-distinctive feature (e.g., *knee*) visible in the target picture (e.g., CAMEL), but no interference for distractor words denoting a distinctive feature (e.g., *hump*) compared to matched sets of distractors denoting unrelated features. We argued this finding is consistent with lexical selection by competition accounts, and might entail additional spread of activation to related concepts that share the non-distinctive feature via the appropriate category node (e.g., Animals). In their commentary, Montefinese and Vinson (2015) arrive at the opposite conclusion, arguing that feature distinctiveness does not affect the degree of interference in PWI. Here, we respond to each of their objections.

Montefinese and Vinson's (2015) principal objection concerned the statistical significance tests of the higher order interaction between distractor relatedness, distinctiveness and stimulus onset asynchrony (SOA), and partial interaction of relatedness and distinctiveness at the −150 ms SOA. Our response to this objection revisits longstanding debates in experimental psychology about the use of arbitrary alpha levels to justify dichotomous decisions and failure to consider the size of effects (e.g., Rosnow and Rosenthal, 1989; Kirk, 2003; Cumming, 2014). In Vieth et al. (2014a), the *p*-values for the higher-order interaction by participants and by items were 0.06 and 0.078, respectively, and the interaction was a medium sized effect (partial η^2^ = 0.10 and 0.11, respectively; e.g., Vacha-Haase and Thompson, 2004). Montefinese and Vinson misrepresent the two-way interaction as being “far from significant.” In fact, this partial interaction was statistically significant in the items analysis and a larger effect (*p* = 0.023; partial η^2^ = 0.21). Surprisingly, Montefinese and Vinson chose to cite Nieuwenhuis et al. (2011) to support their criticism of the statistical significance tests of the interactions, yet those authors wrote: “as famously noted by Rosnow and Rosenthal, ‘surely, God loves the 0.06 nearly as much as the 0.05.’ Thus, when making a comparison between two effects, researchers should report the statistical significance of their difference rather than the difference between their significance levels.” (p. 1105). This is precisely the approach taken in Vieth et al. (2014a): interactions were reported and an arbitrary 0.05 level was not used to justify a dichotomous decision (cf. Montefinese and Vinson, 2015).

Of note, other researchers have followed marginally significant interactions with planned comparisons in PWI studies and offered discussions about why higher order interactions are particularly difficult to detect (e.g., Starreveld and La Heij, 1995; Damian and Martin, 1999). This two-pronged approach is preferred because semantic relatedness effects (i.e., interference and facilitation) in PWI are defined and demonstrated by planned comparisons of related vs. unrelated distractors. Costa et al. (2005) reported facilitation with related feature distractors using this approach. In Vieth et al. (2014b) we likewise contrasted distinctive and non-distinctive feature distractors with their respective matched unrelated distractor sets, as these were the chief comparisons we were interested in examining *prior to* commencing data collection. We were unable to create perfectly matched sets of distinctive and non-distinctive distractors, as we noted. Hence, our hypotheses applied to only a subset of the data contributing to the interaction term, as there was no a priori reason for contrasting the two *unrelated* distractor sets. Only non-distinctive feature distractors showed a significant interference effect both by participants (*p* = 0.011) and by items (*p* = 0.003), of moderate to large effect size (*d* = 0.52 and 0.69, respectively). It is worth emphasizing planned contrasts are typically conducted *whether or not the overall F-test is significant*; at least this is the convention according to introductory experimental design and statistics textbooks (e.g., Kirk, 1982; Rosenthal and Rosnow, 1985; Keppel, 1991; Lomax and Hahs-Vaughn, 2012).

Montefinese and Vinson (2015) next expressed concern about Vieth et al.'s failure to match distinctive and non-distinctive features across distractor sets in terms of a “crucial semantic variable” called *dominance*—or production frequency, noting that many of the non-distinctive distractors (e.g., *bone, skin*) were not listed in the McRae et al.'s (2005) feature production norms. Production frequency refers to the number of participants that list a particular feature for a given concept in feature norming tasks, and has been interpreted as a measure of semantic saliency (e.g., Cree et al., 2006). Montefinese and Vinson argued that as many of the non-distinctive distractors were not listed in the McRae et al. norms, they were not salient for their related target concepts, and therefore were not sufficiently strong lexical competitors to induce the significant interference effect that we observed. As this argument relies on the novel assumption that dominance is a crucial measure of the strength of conceptual feature activation in PWI, it is worth examining in more detail.

For pragmatic purposes, researchers impose limits on both the time allowed and on the number of features to be produced for a given concept in feature norming studies, and then only include features in the resulting norms if a minimum number of participants listed them (e.g., 5/30 in McRae et al.'s, 2005). It is not surprising then, that feature norms do not represent exhaustive listings for each concept. Indeed as Montefinese et al. (2014) put it: “in a (theoretically) ideal experiment, if we asked a group of participants to list all of the possible features of a given concept, without posing any temporal or quantitative limits, we would end having a very large number of features with a dominance value close or equal to 1. In fact, even the less important feature for that concept would eventually be listed, by most of the participants, *making dominance a poor measure of a feature's importance/salience for a concept representation*.” (italics added; p. 356–357). We concur with Montefinese et al. (2014) view.

As Montefinese and Vinson (2015) noted, we were careful to match our PWI stimuli on a range of variables. In particular, we ensured associative relations between distractors and targets were minimal (Nelson et al., 2004). In free-association tasks, participants are given a cue word and required to list words that come to mind, a substantial proportion of which are features (De Deyne and Storms, 2008). Thus, “free association probabilities index the relative accessibility of related words in memory” (Nelson et al., 2004, p. 402). It should be apparent that dominance and free association probability are both measures of the relative availability of lexical knowledge. The potential collinearity between the two measures was acknowledged by Montefinese et al. (2014): “Consequently, if we consider the feature listing as a type of controlled word association task wherein the words are restricted to the features (Cree et al., 2006), we could presume that the order of production would also capture the lexical association link between concept and feature.” (pp. 366–367). It therefore should not come as a surprise that the majority of our distractor stimuli in Vieth et al. (2014a) had low dominance, because features with high free association probabilities/high production frequencies had been mostly eliminated during their construction. However, the correlation is not perfect. When presented with the cue CAMEL, only a small proportion (22/148 or 15%) of the participants in the Nelson et al. (2004) normative study freely associated the concept with the feature *hump*, indicating it was not particularly accessible or salient to them. As Montefinese and Vinson point out, a higher proportion listed *hump* in McRae et al. (2005) normative study due to the use of an explicit instruction to list *only* the features of a CAMEL. Of the two measures, our view is that lexical association is the more important variable to control, as it has already been shown to influence naming latencies in PWI experiments.

Further, there is no evidence to support Montefinese and Vinson's claim of “substantial differences” in dominance of the distinctive and non-distinctive features in Vieth et al. The median production frequency for our distinctive distractors was low, only 8/30 (or 27%) according to McRae et al.'s (2005) norms. While dominance has been shown to influence decision latencies on feature verification tasks, the classic studies showing these effects contrasted *high* (i.e., greater than 50%) vs. low production frequencies (e.g., Conrad, 1972; Ashcraft, 1978). Feature verification, in which participants are asked to indicate whether given features are true of target concepts (e.g., by reading property statements), also differs considerably from instructing participants to name a target picture while ignoring a distractor word. This difference in procedures is important to emphasize, as it is well-established that semantic relatedness effects depend critically on the nature of the experimental task. Given all of the above, we find little reason to entertain Montefinese and Vinson's conjecture about dominance significantly influencing our findings.

Aside from dominance, another theoretical issue introduced by Montefinese and Vinson (2015) in their commentary is the level-of-specificity between distractor and target in PWI. For example, (Hantsch et al., 2005; also Hantsch and Mädebach, 2013) reported that subordinate level distractors (e.g., *Mini*) produced interference when related objects were named at the basic level (e.g., CAR). Montefinese and Vinson (2015) speculate our findings might be akin to a level-of-specificity effect and propose that the presence of the feature denoted by the distractor in the target picture (e.g., *knee*) might “permit further activation of its name as a potentially plausible alternative to the basic-level target name” (e.g., CAMEL).

Montefinese and Vinson do not provide an adequate explanation of the mechanism by which merely raising the lexical activation level of a feature-level distractor representation would make it a plausible response when the instruction is to name the entire target object (e.g., in Hantsch et al.'s level-of-specificity manipulation above, Mini and CAR are appropriate names for a picture of a Mini as they both denote the entire object, unlike *wheel*). We note the similarity of this proposal with Mahon et al.'s (2007) response exclusion account that attributes semantic interference to a post-lexical decision mechanism operating according to response-relevant criteria. As we acknowledged in Vieth et al. (2014b), the response exclusion account could possibly be modified to explain our finding by assuming visible features of target pictures constitute response relevant criteria, despite the instruction to name the whole object. However, this would involve abandoning Mahon et al.'s (2007) assumption that conceptual feature overlap does not constitute a response-relevant criterion.

Montefinese and Vinson's (2015) more empirical objection to our finding of semantic interference concerned the −150 ms SOA at which the significant effect was observed in Experiment 3. Specifically, they characterized the result as “temporally-selective,” implying the effect should have been observed at both 0 and −150 ms SOAs in the one experiment. In PWI timecourse studies, the semantic interference effect for category coordinates is typically found at either −150 or 0 ms SOAs, yet the literature shows the effect is often *not* significant at both of these SOAs within the same experiment (e.g., La Heij et al., 1990; Schriefers et al., 1990; Starreveld and La Heij, 1996; Damian and Martin, 1999; Damian and Bowers, 2003). In Sailor and Brooks' (2014) PWI study, significant interference from feature distractors was observed at the 0 ms SOA but not at −150 ms. This variability in the reported results for early and 0 ms SOAs likely reflects procedural differences across PWI studies, such as the use of written vs. auditory distractors, or central vs. random positioning of written distractors. In the PWI procedure, auditory distractors take longer to process than written ones, so an interference effect is more likely to be obtained at earlier SOAs (see Damian and Martin, 1999). Less-predictable placement of distractor words will likewise affect processing time. Consequently, there is no empirical reason to accept Montefinese and Vinson's stipulation that significant interference for related feature distractors should occur at both SOAs in the one PWI experiment.

According to the lexical selection by competition account, semantic interference is the result of a tradeoff between the priming of the target representation by the distractor and the priming of the distractor's lexical representation by the target (e.g., Roelofs, 1992). Montefinese and Vinson (2015) objected to our adopting this interpretation for the significant interference effect at −150 ms (e.g., Roelofs, 1992; Starreveld and La Heij, 1996). Their premise is that activation cannot spread from target to distractor at the −150 ms SOA as the target picture is yet to be presented. Logically, this criticism would also apply to the reported findings of semantic interference for category-coordinates at −150 ms and earlier SOAs, and so challenge lexical-selection-by competition accounts, if the premise were accurate (e.g., La Heij et al., 1990; Schriefers et al., 1990; Starreveld and La Heij, 1995, 1996; Damian and Martin, 1999). However, Montefinese and Vinson's (2015) premise reflects a misunderstanding of the typical PWI procedure that involves written distractor words remaining on screen while the target picture is presented and until the participant responds (e.g., Vieth et al., 2014a). As naming latencies in PWI are usually between 600 to 800 ms, this provides ample time for target-to-distractor priming.

In conclusion, we find none of the theoretical, empirical, or statistical objections raised by Montefinese and Vinson (2015) to be particularly compelling, as they reflect a combination of conjecture, misunderstanding and misrepresentation. However, we concur with them that the details of conceptual representations remain underspecified in production models. To this end, further empirical work is needed, rather than commentary.

## Funding

This work was supported by Australian Research Council Discovery Project Grants DP1092619 and DP150103997.

### Conflict of interest statement

The authors declare that the research was conducted in the absence of any commercial or financial relationships that could be construed as a potential conflict of interest.
